# Supplementary material for: How many markers are needed to robustly determine a cell's type?
Source: iScience. 2021 Oct 14;24(11):103292. doi: 10.1016/j.isci.2021.103292 (PMC8571500; doi:10.1016/j.isci.2021.103292)
Supplement: Document S1. Figures S1–S9 [file mmc1.pdf]

iScience, Volume *24*

Supplemental information

How many markers are needed

to robustly determine a cell's type?

Stephan Fischer and Jesse Gillis

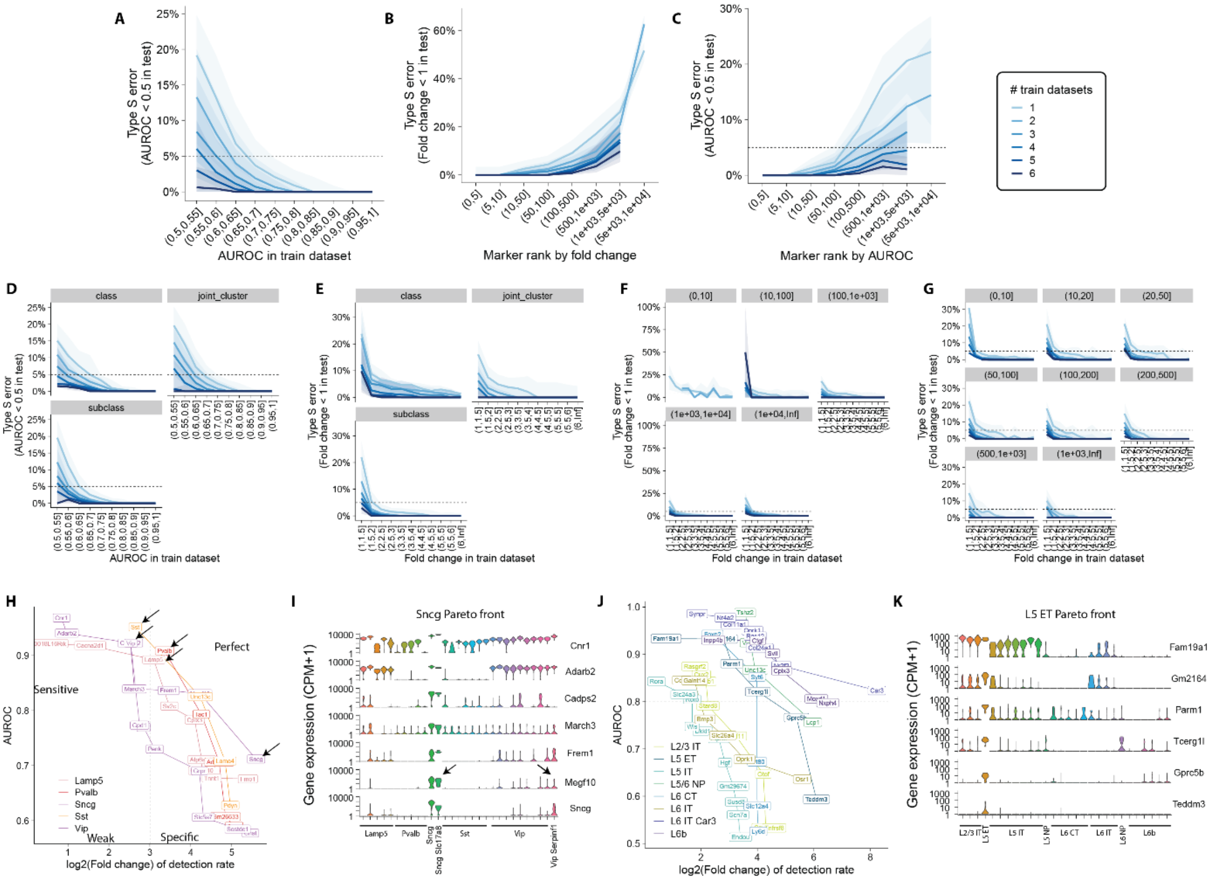

Figure S1. Replicable DE thresholds and subclass Pareto markers, related to Figure 1. A-C Type S error as a function of AUROC in train datasets (A), marker rank by fold change (B) and marker rank by AUROC (C). The dashed line indicates a type S error of 5%, ribbons around lines indicate variability across cell types and test datasets. **D-G** Type S error as a function of AUROC (D) or FC (E-G) in train dataset, with facets showing variability across hierarchy level (D,E), average cell type size (F) and average gene expression (G). **H** Pareto fronts in FC/AUROC space for inhibitory subclasses. Arrows point to the main historical marker for each subclass. **I** Expression of genes on the Sncg Pareto front across BICCN inhibitory clusters. **J** Pareto fronts in FC/AUROC space for excitatory subclasses. **K** Expression of genes on the L5 ET Pareto front across BICCN excitatory clusters.

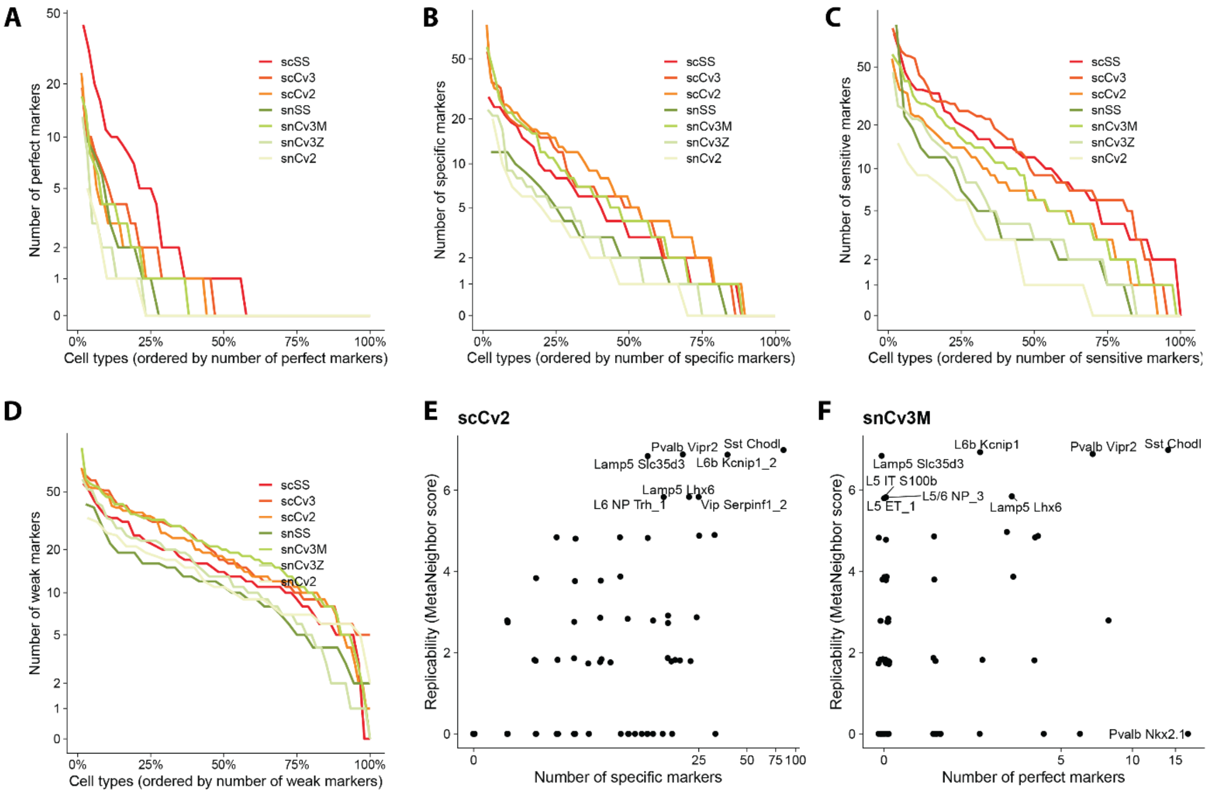

Figure S2. Number of markers and cell type replicability at the highest cell type resolution, related to Figure 2. A-D Number of perfect markers (A), specific markers (B), sensitive markers (C), and weak markers (D) for BICCN clusters, with cell types ordered according to number of markers, colored according to the dataset used to compute markers. **E-F** MetaNeighbor replicability as a function of the number of specific markers in the scCv2 dataset (E) and the number of perfect markers in the snCv3M dataset (F).

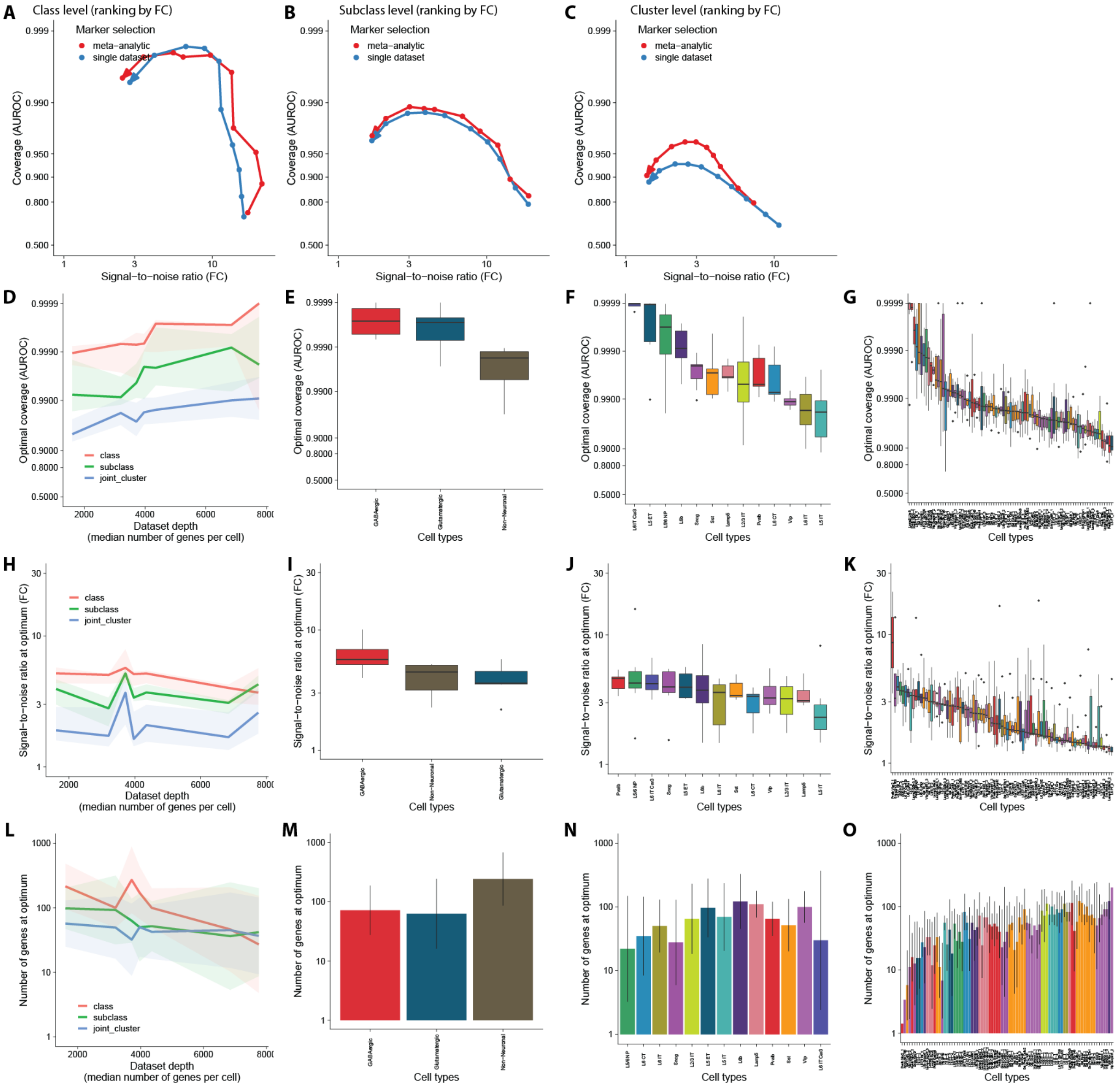

Figure S3. Optimal number of genes, AUROC and Fold Change for BICCN cell types, related to Figure 3. **A-C** Parametric curve in FC/AUROC space showing evolution of classification performance with an increasing number of marker genes at the class (A), subclass (B) and joint cluster (C) level. **D-G** Breakdown of optimal AUROC performance (meta-analytic markers) as a function of dataset depth, colored by hierarchy level (D), for individual classes, showing variability across test datasets (E), for individual subclasses, showing variability across test datasets (F), for individual clusters, showing variability across test datasets (G). **H-K** Same as D-G with signal-to-noise ratio (FC) at optimal performance instead of AUROC. **L-O** Same as D-G with number of genes at optimal performance instead of AUROC.

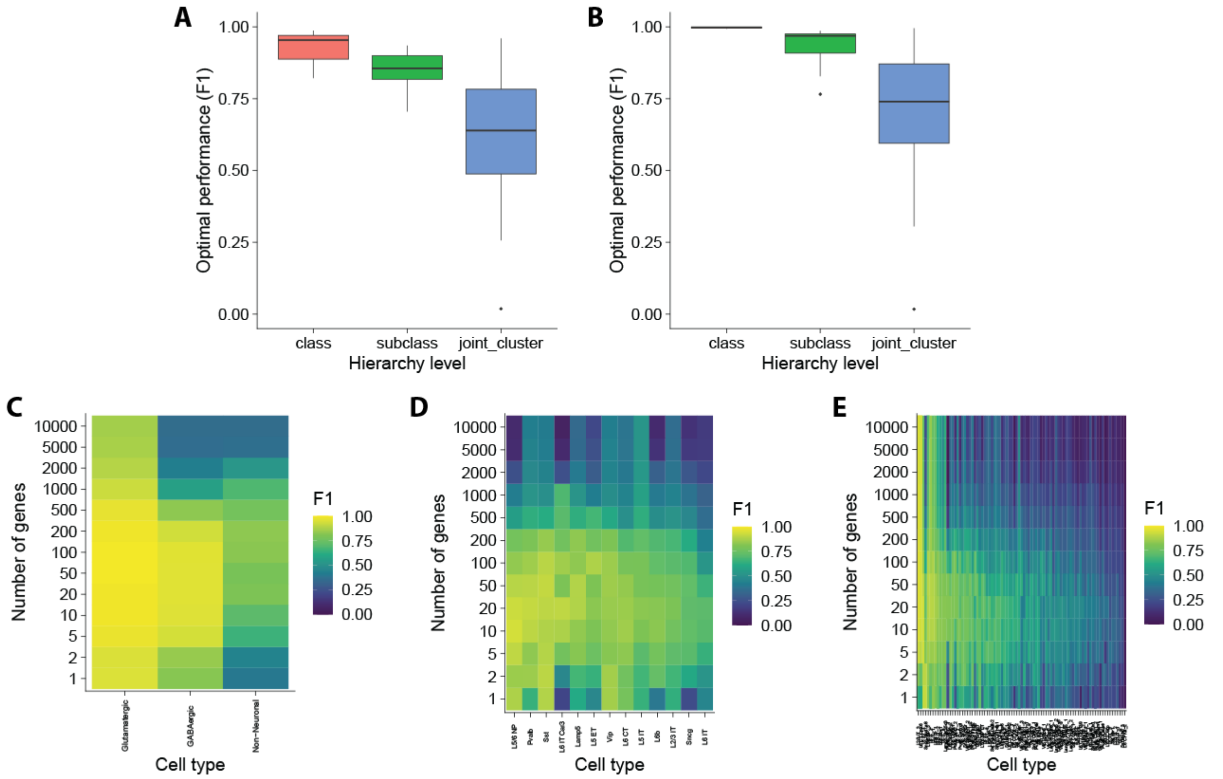

Figure S4. Classification performance with transcriptome-wide normalization, related to Figure 4. **A-B** Summary of optimal classification performance (F1) across hierarchy levels with transcriptome-wide normalization (A) and marker-wide renormalization (B). Variability is shown across cell types and test datasets. **C-E** Heatmap detailing classification performance for each cell type as a function of the number of genes at the class (C), subclass (D) and cluster (E) level.

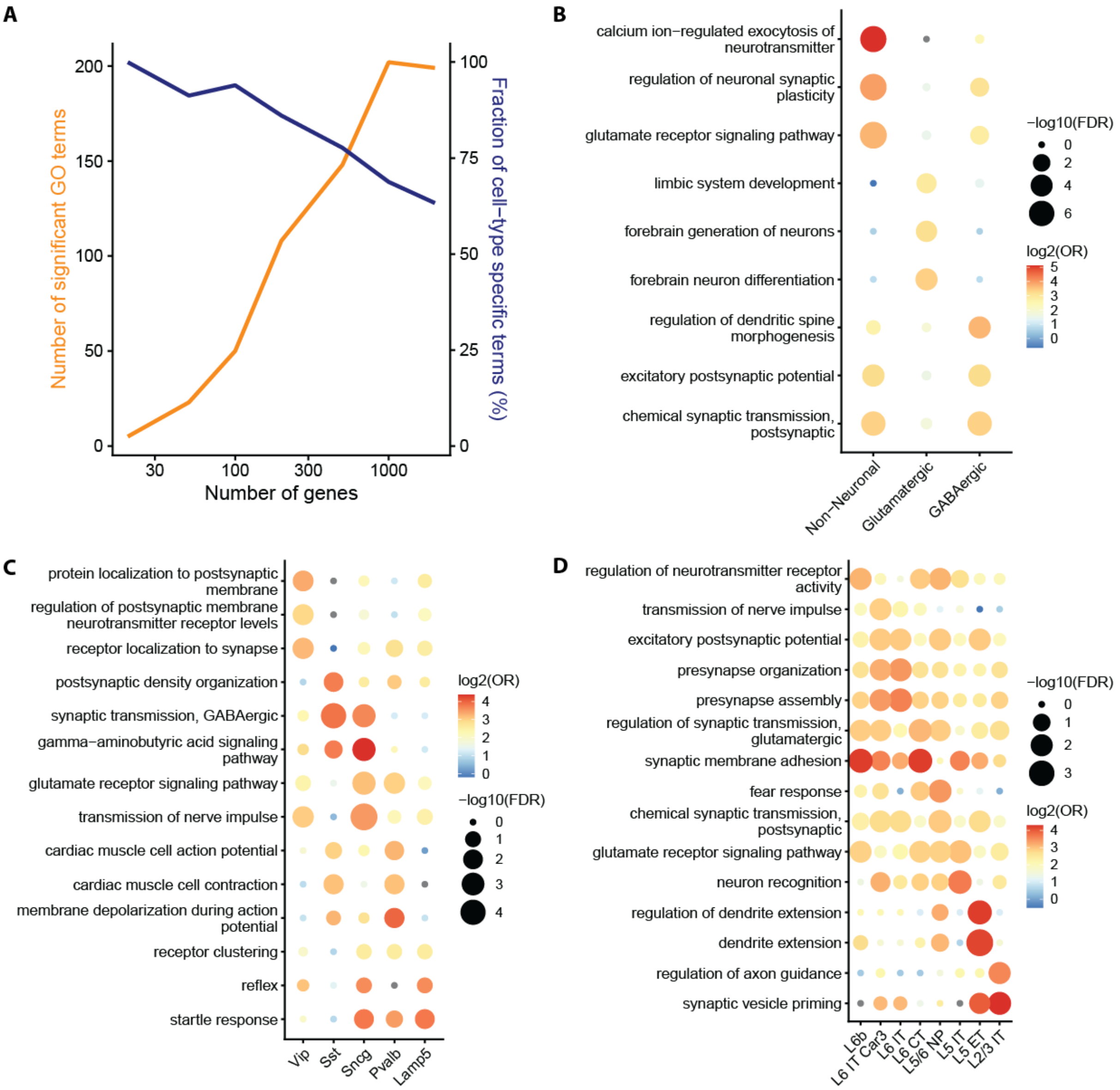

Figure S5. Top 200 meta-markers show strong, but less specific, enrichment for synaptic processes, related to Figure 5. A Total number of significantly enriched GO terms (orange) and fraction of significant GO terms that are enriched in a unique cell type (blue) for BICCN classes when an increasing number of meta-markers are considered. **B** Top 3 enriched Gene Ontology (GO) terms for the top 200 meta-markers for each BICCN class. For each dot, the size reflects the False Discovery Rate (FDR), the color reflects the Odds Ratio (OR) of the enrichment test (hypergeometric test). **C** Same as B for the top 200 meta-markers for BICCN GABAergic subclasses. **D** Same as B for the top 200 meta-markers for BICCN Glutamatergic subclasses (only top 2 terms are shown).

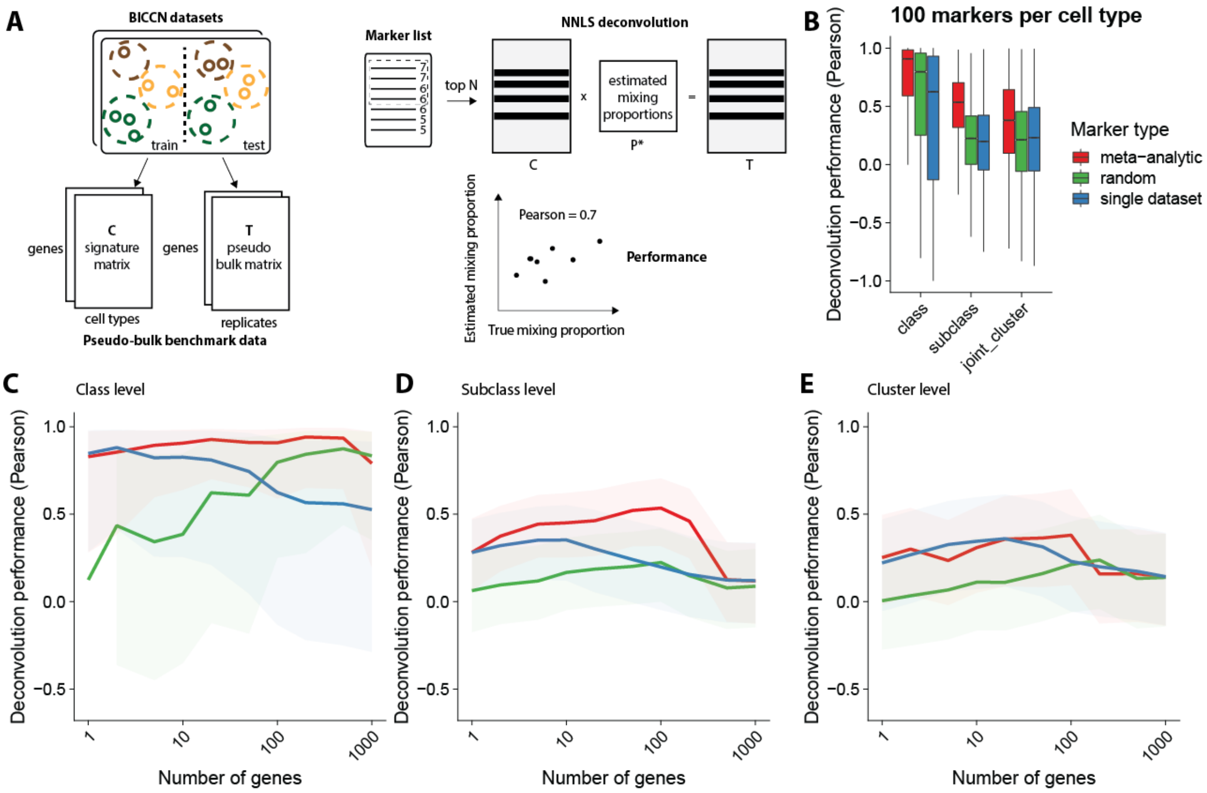

Figure S6. Meta-analytic markers improve deconvolution performance at every level of the hierarchy, related to STAR Methods. A Schematic of deconvolution task. **B** Summary of deconvolution performance (Pearson's r) at each hierarchy level with 100 markers per cell type. Colors show 3 marker prioritization strategies (single dataset markers, meta-analytic markers or expression-level matched random genes). **C-E** Deconvolution performance (Pearson correlation of true and estimated cell type proportions) for 3 marker prioritization strategies at the class level (C), the subclass level (D), and the cluster level (E). Colors as B.

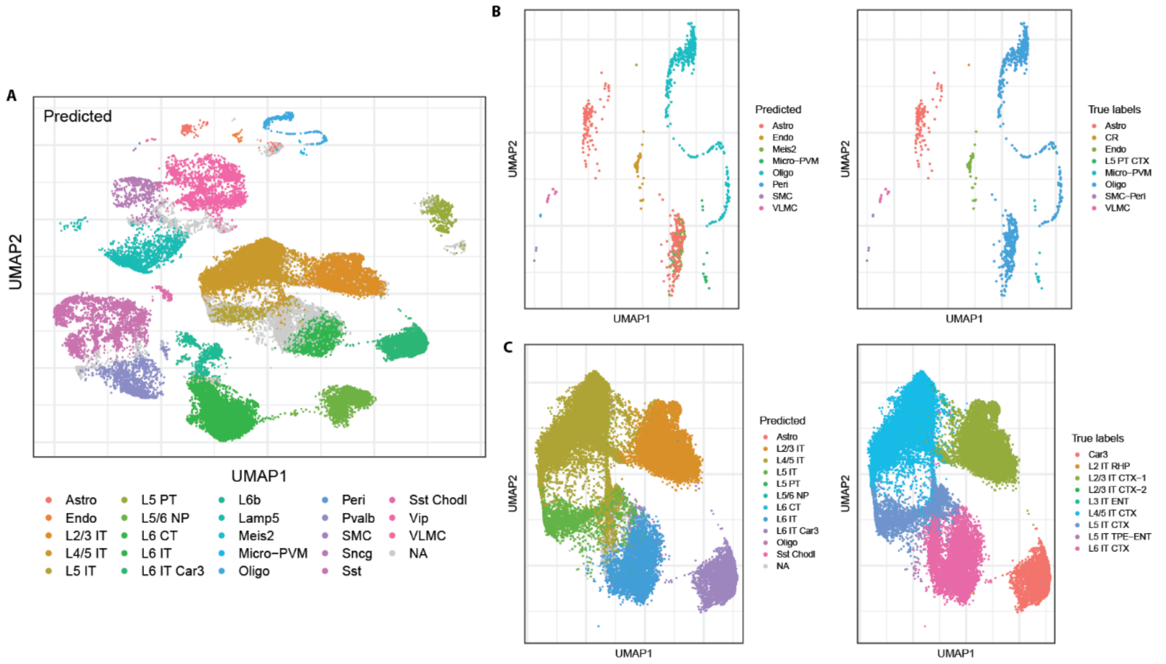

Figure S7. Focus on subclass-level predictions in the auditory cortex, related to Figure 6. A Subclass-level predictions in the auditory cortex based on the top 100 meta-markers. Cells remain unassigned (NA) if the enrichment score is lower than 2 for all subclasses. **B** Subclass-level predictions for non-neurons in the auditory cortex based on the top 100 meta-markers (left) and reference labels (right). **C** Subclass-level predictions for Intra-Telencephalic (IT) excitatory neurons in the auditory cortex based on the top 100 meta-markers (left) and reference labels (right).

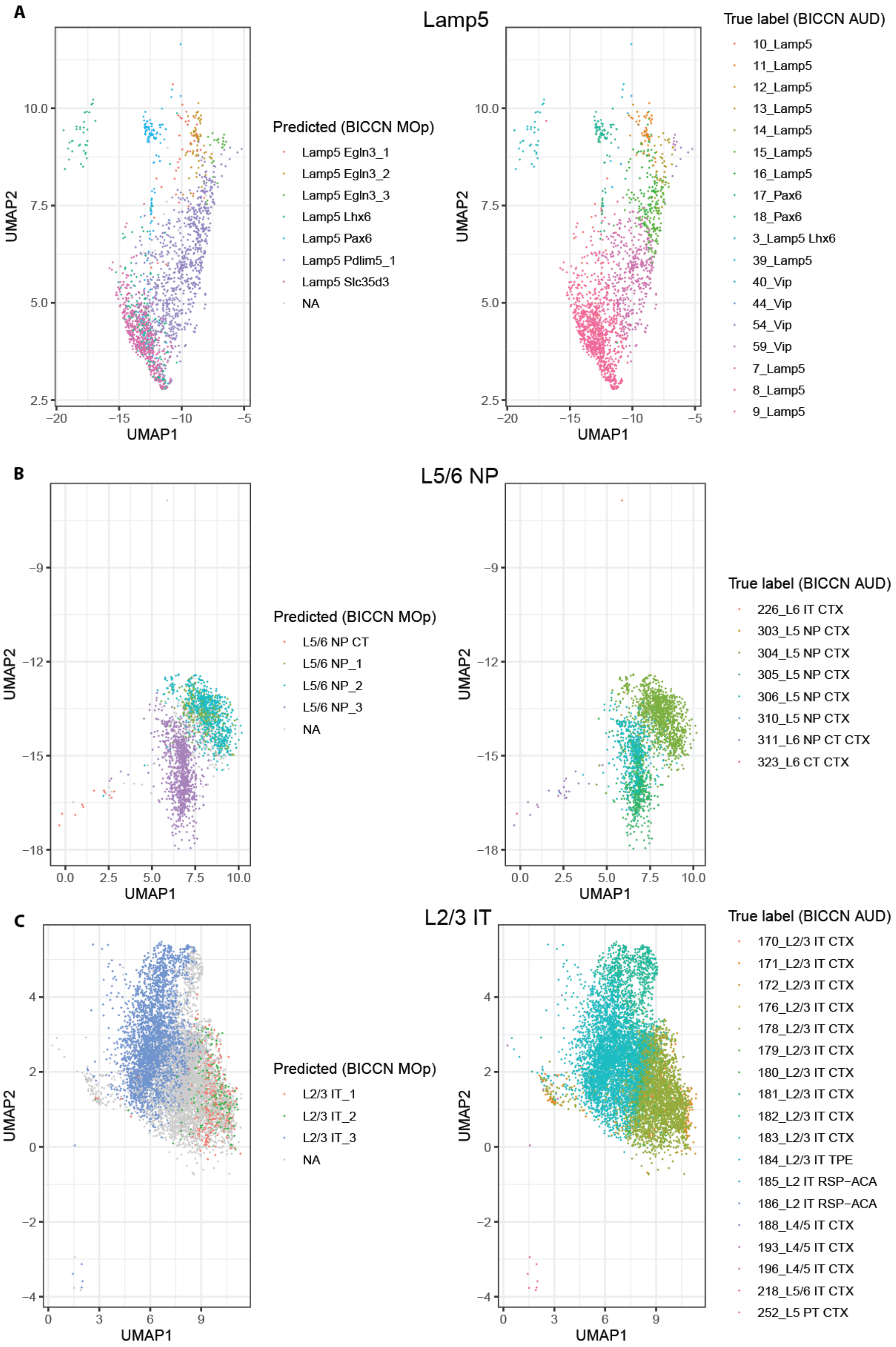

Figure S8. Cluster-level predictions in the auditory cortex, related to Figure 6. A-C Cluster-level predictions for Lamp5 inhibitory neurons (A), Near-Projecting (NP) excitatory neurons (B) and layer 2/3 Intra-Telencephalic (IT) excitatory neurons (C) in the auditory cortex based on the top 100 meta-markers (left) and reference labels (right). In all panels, cells remain unassigned (NA) if the enrichment score is lower than 1.5 for all clusters.

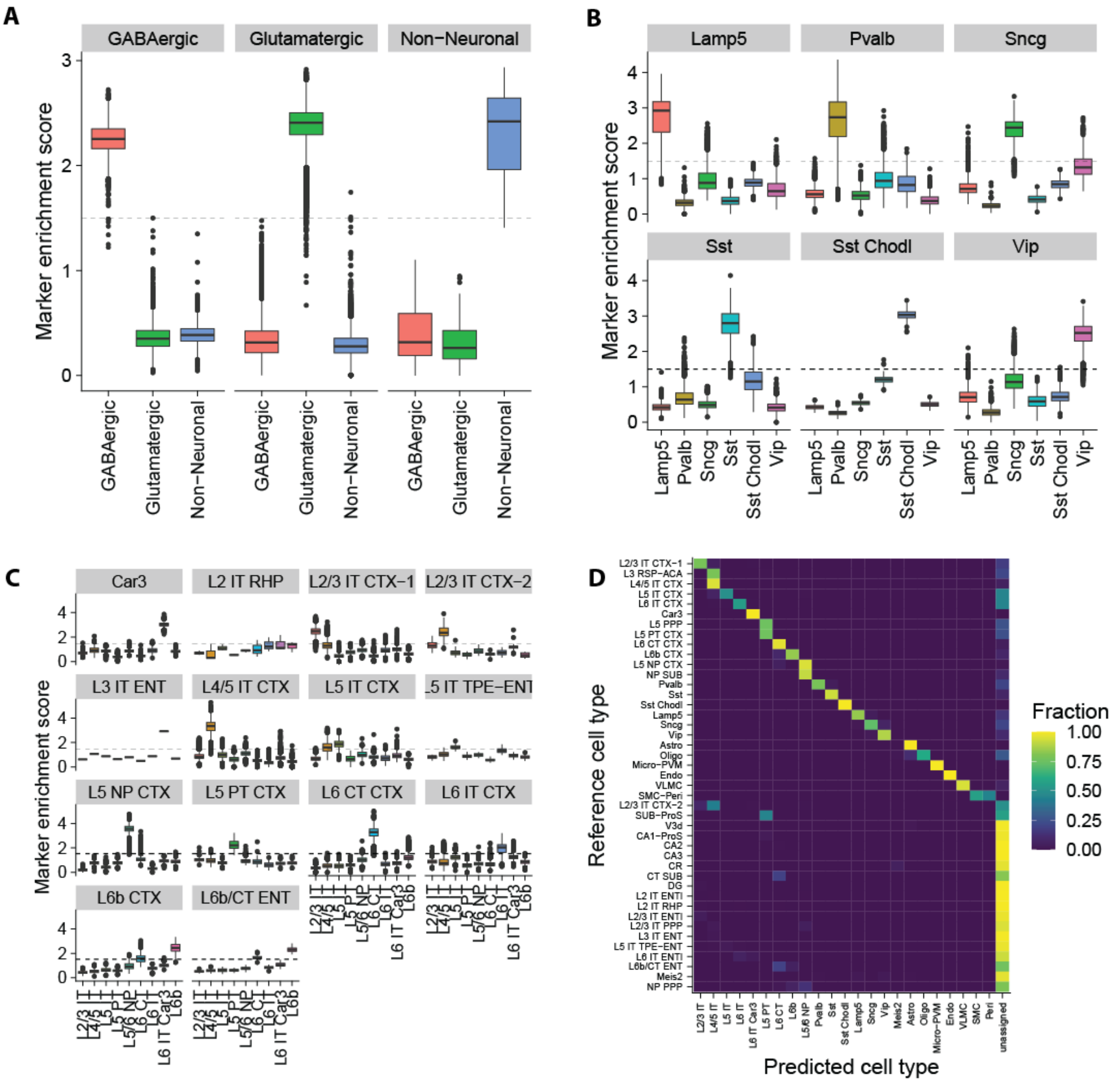

Figure S9. The marker enrichment score provides robust separability of cell types in other cortical regions, related to Figure 6. A Marker enrichment scores based on the top 100 meta-markers for the 3 BICCN classes in the auditory cortex. The facets are organized according to reference cell types (from the auditory cortex), the x-axis according to meta-markers sets (for the motor cortex). **B** Same as A for BICCN inhibitory subclasses. **C** Same as A for BICCN excitatory subclasses. **D** Confusion matrix showing the concordance of subclass-level predictions based on the top 100 meta-markers with reference cell types across 40 brain areas. Cells are unassigned if the marker enrichment is lower than 2 for all subclasses.
